# Supplementary material for: In Silico Characterization of the Secretome of the Fungal Pathogen Thielaviopsis punctulata, the Causal Agent of Date Palm Black Scorch Disease
Source: J Fungi (Basel). 2023 Feb 27;9(3):303. doi: 10.3390/jof9030303 (PMC10051545; doi:10.3390/jof9030303)
Supplement: Supplementary file 1 [file jof-09-00303-s001.zip › Supplementary Table S3.pdf]

Table S3. Overview of CAZymes in the secretome of *Thielaviopsis* species

CAZyme Family	*Thielaviopsis* species					
	T. punctulata	*T. ethacetica*	*T. cerberus*	*T. euricoi*	*T. musarum*	*T. populi*
AA1	KKA29108.1	Test_03282-RA	T.cer_02404-RA	T.eur_03837-RA	Tmus_06880-RA	T.pup_01818-RA
	KKA30055.1			T.eur_04384-RA		T.pup_02029-RA
	KKA28993.1					
AA16	KKA29756.1					
AA2	KKA28039.1			T.eur_02297-RA		
	KKA29461.1					
AA3	KKA28521.1	Test_02462-RA		T.eur_00155-RA	Tmus_06232-RA	T.pup_05686-RA
	KKA30608.1	Test_02495-RA		T.eur_00941-RA	Tmus_06707-RA	
	KKA31082.1	Test_05836-RA		T.eur_00974-RA		
AA5	KKA28638.1	Test_06742-RA	T.cer_04144-RA	T.eur_01371-RA	Tmus_05249-RA	T.pup_01027-RA
AA7	KKA27659.1				Tmus_04258-RA	
	KKA30937.1				Tmus_06194-RA	
AA8		Test_01779-RA	T.cer_01944-RA	T.eur_01478-RA		T.pup_02050-RA

						T.pup_02231-RA
AA9	KKA27328.1	Test_01326-RA		T.eur_02013-RA	Tmus_00287-RA	T.pup_02046-RA
	KKA28212.1	Test_01766-RA		T.eur_06378-RA	Tmus_02364-RA	T.pup_04518-RA
	KKA28497.1	Test_03489-RA		T.eur_06380-RA	Tmus_03461-RA	T.pup_04520-RA
	KKA29219.1	Test_03491-RA		T.eur_06526-RA	Tmus_06701-RA	
	KKA25994.1	Test_04367-RA		T.eur_06579-RA	Tmus_06703-RA	
	KKA29038.1					
	KKA25992.1					
AA11		Test_03372-RA	T.cer_02164-RA	T.eur_02331-RA	Tmus_03171-RA	T.pup_02374-RA
		Test_03859-RA		T.eur_02512-RA	Tmus_03228-RA	
AA12		Test_04402-RA				
CE1		Test_02988-RA	T.cer_02955-RA	T.eur_02141-RA	Tmus_00844-RA	T.pup_02171-RA
				T.eur_05985-RA	Tmus_03737-RA	
CE3		Test_03304-RA				
CE4	KKA26186.1	Test_01310-RA	T.cer_05232-RA	T.eur_05828-RA		
	KKA27343.1					

CE5	KKA30377.1	Test_03741-RA	T.cer_04406-RA	T.eur_05948-RA	Tmus_00818-RA	
	KKA30382.1		T.cer_04567-RA			
GH03	KKA26832.1	Test_02208-RA	T.cer_01805-RA	T.eur_01772-RA	Tmus_01989-RA	
	KKA30767.1	Test_02663-RA	T.cer_05853-RA	T.eur_04142-RA		
GH05					Tmus_01003-RA	T.pup_00268-RA
	KKA28137.1	Test_05858-RA	T.cer_06171-RA			
	KKA26007.1					
	KKA26778.1					
GH06				T.eur_06053-RA		
GH07	KKA26295.1	Test_01774-RA	T.cer_02909-RA		Tmus_05285-RA	
	KKA28489.1					
GH10	KKA27891.1	Test_01612-RA	T.cer_04740-RA	T.eur_00342-RA	Tmus_00703-RA	T.pup_04970-RA
	KKA29568.1			T.eur_03277-RA	Tmus_04890-RA	
GH11	KKA29107.1	Test_02894-RA	T.cer_00211-RA	T.eur_02105-RA	Tmus_03052-RA	T.pup_02434-RA
	KKA30007.1	Test_03021-RA	T.cer_00248-RA	T.eur_02230-RA	Tmus_05573-RA	

		Test_05896-RA	T.cer_04430-RA	T.eur_04262-RA		
GH12			T.cer_05248-RA			
GH13						T.pup_03937-RA
GH115	KKA28239.1					
GH125	KKA28305.1					
GH128	KKA29105.1					
GH13	KKA30803.1					
GH131	KKA28951.1			T.eur_00151-RA		T.pup_01834-RA
	KKA29646.1					
GH132	KKA26122.1					
GH15	KKA29558.1					T.pup_01221-RA
GH16	KKA30944.1	Test_01549-RA	T.cer_05003-RA	T.eur_00403-RA	Tmus_04715-RA	T.pup_03572-RA
	KKA27451.1	Test_02225-RA	T.cer_04189-RA	T.eur_05243-RA	Tmus_01083-RA	T.pup_01931-RA
	KKA26151.1	Test_04541-RA	T.cer_05132-RA	T.eur_06909-RA	Tmus_02351-RA	T.pup_05073-RA
	KKA28499.1	Test_06379-RA	T.cer_03925-RA	T.eur_06418-RA		
		Test_06873-RA	T.cer_05758-RA	T.eur_06503-RA		

GH17		Test_06398-RA	T.cer_04669-RA	T.eur_02647-RA	Tmus_05762-RA	T.pup_04902-RA
	KKA27515.1	Test_00795-RA	T.cer_02087-RA	T.eur_02730-RA	Tmus_00656-RA	T.pup_00172-RA
	KKA30054.1	Test_03483-RA	T.cer_02495-RA	T.eur_03840-RA	Tmus_04290-RA	T.pup_03210-RA
	KKA30697.1		T.cer_02547-RA		Tmus_04319-RA	T.pup_04965-RA
	KKA26416.1					
GH20	KKA30299.1	Test_02221-RA	T.cer_06535-RA	T.eur_04129-RA	Tmus_01079-RA	T.pup_00618-RA
GH28	KKA31208.1		T.cer_05134-RA			
GH30	KKA27339.1	Test_01303-RA	T.cer_03773-RA	T.eur_05822-RA	Tmus_02572-RA	
	KKA29858.1	Test_01905-RA				
GH31					Tmus_00763-RA	
GH32	KKA28220.1	Test_01348-RA	T.cer_06473-RA	T.eur_05865-RA	Tmus_06395-RA	T.pup_03291-RA
GH37	KKA30799.1		T.cer_00448-RA			
GH38	KKA26248.1					
GH43	KKA28970.1	Test_04417-RA	T.cer_00615-RA	T.eur_06953-RA	Tmus_05267-RA	T.pup_05151-RA
	KKA29859.1	Test_01906-RA	T.cer_07857-RA	T.eur_02063-RA	Tmus_02571-RA	T.pup_02166-RA
	KKA30545.1	Test_06941-RA	T.cer_06416-RA	T.eur_02859-RA	Tmus_06751-RA	T.pup_01537-RA

	KKA27803.1	Test_04823-RA	T.cer_03683-RA	T.eur_04875-RA		
				T.eur_05513-RA		
GH45	KKA28018.1					
GH51	KKA29147.1					
GH53	KKA29651.1					T.pup_04245-RA
GH55	KKA30026.1	Test_05771-RA	T.cer_02346-RA	T.eur_04781-RA		T.pup_02839-RA
	KKA30509.1					T.pup_05194-RA
GH63		Test_00128-RA		T.eur_00572-RA	Tmus_04737-RA	
GH64	KKA27366.1					
GH72		Test_02198-RA	T.cer_02621-RA	T.eur_04152-RA	Tmus_05081-RA	T.pup_00010-RA
			T.cer_03435-RA	T.eur_04630-RA	Tmus_05337-RA	T.pup_00923-RA
						T.pup_04546-RA
GH74			T.cer_02329-RA	T.eur_06581-RA		
GH76	KKA26192.1	Test_00116-RA	T.cer_01044-RA	T.eur_00560-RA		T.pup_00396-RA
		Test_04990-RA	T.cer_01269-RA	T.eur_04963-RA	Tmus_02078-RA	T.pup_06102-RA
					Tmus_04908-RA	
GH78	KKA27604.1		T.cer_01031-RA			

GH92		Test_04019-RA		T.eur_02770-RA	Tmus_03757-RA	T.pup_00920-RA
GH93	KKA30496.1	Test_03146-RA			Tmus_06638-RA	
GT4	KKA26489.1					
GT8				T.eur_04896-RA		T.pup_02843-RA
GT32						T.pup_04652-RA
GT34		Test_00049-RA		T.eur_00490-RA	Tmus_00078-RA	
GT61		Test_02790-RA				
PL1	KKA26877.1	Test_05522-RA	T.cer_02030-RA	T.eur_02387-RA	Tmus_06398-RA	T.pup_03027-RA
	KKA27238.1	Test_06829-RA		T.eur_06641-RA		T.pup_03895-RA
PL3	KKA30830.1	Test_05658-RA		T.eur_05722-RA		T.pup_04517-RA
PL4	KKA28462.1					T.pup_05359-RA
