# Supplementary material for: In Silico Characterization of the Secretome of the Fungal Pathogen Thielaviopsis punctulata, the Causal Agent of Date Palm Black Scorch Disease
Source: J Fungi (Basel). 2023 Feb 27;9(3):303. doi: 10.3390/jof9030303 (PMC10051545; doi:10.3390/jof9030303)
Supplement: Supplementary file 1 [file jof-09-00303-s001.zip › Supplementary Table S4.pdf]

Supplementary Table S4. Secretory proteases in Six Thielaviopsis species

Protease Family	Protease Family member	PFAM domain	*T. punctulata* Protein_ID	*T. cerberus* Protein_ID	*T.musarum* Protein_ID	*T.ethacetica* Protein_ID	*T.euricoi* Protein_ID	*T.populi* Protein_ID
Aspartic protease	Asp_2	PF00026.26	KKA30396.1	T.cer_06062-	Tmus_03923-RA	Test_06399-		T.pup_0189
		PF00026.26	KKA30997.1	T.cer_05252-	Tmus_05761-RA	Test_07058-		T.pup_0370
		PF00026.26	KKA30325.1	T.cer_04668-	Tmus_04944-RA	Test_03944-		T.pup_03666
	Peptidase_A4	PF01828.20	KKA31244.1		Tmus_06792-RA	Test_03342-		
Carboxy	Peptidase_C13	PF01650.21	KKA30601.1	T.cer_00703-	Tmus_06059-RA	Test_01795-	T.eur_06606-RA	T.pup_04166
	Peptidase_C19	PF01650.21		T.cer_07956-	Tmus_04466-RA	Test_06152-		T.pup_0510
Metallo protease	Peptidase_M14	PF00246.27	KKA28028.1	T.cer_03215-	Tmus_00763-RA	Test_06184-		
		PF00246.27	KKA28562.1			Test_02270-		
	Peptidase_M20	PF01546.31			Tmus_04757-RA	Test_04347-		T.pup_02179
	Peptidase_M28	PF04389.20	KKA26478.1	T.cer_03724-RA	Tmus_00266-RA	Test_04128-		T.pup_0358
		PF04389.20	KKA27661.1		Tmus_03937-RA			
	Peptidase_M36	PF02128.18	KKA27194.1		Tmus_04757-RA			
	Peptidase_M43	PF05572.16	KKA26166.1	T.cer_07857-	Tmus_05267-RA			
Serine petidase	Peptidase_S8	PF00082.25	KKA26451.1	T.cer_05301-RA	Tmus_06699-RA	Test_04445-	T.eur_02394-RA	T.pup_04575
		PF00082.25	KKA27603.1	T.cer_01257-RA	Tmus_00079-RA	Test_06823-	T.eur_02739-RA	T.pup_01758
		PF00082.25	KKA27995.1	T.cer_00335-	Tmus_03525-RA	Test_01877-	T.eur_02884-RA	T.pup_0230
		PF00082.25	KKA29831.1		Tmus_02599-RA	Test_06091-	T.eur_06382-RA	T.pup_06089
		PF00082.25	KKA30987.1		Tmus_00663-RA	Test_03988-	T.eur_00167-RA	
		PF00082.25	KKA30584.1		Tmus_06403-RA	Test_03070-	T.eur_03473-RA	
		PF00082.25	KKA27233.1		Tmus_00996-RA			
		PF00082.25	KKA28625.1					
		PF00082.25	KKA26827.1					
		PF09286.14	KKA31088.1					
	Peptidase_S9	PF00326.24		T.cer_02955-RA	Tmus_03737-RA	Test_02988-	T.eur_02141-RA	T.pup_02171
		PF00326.24		T.cer_04113-	Tmus_00844-RA	Test_02088-		
		PF00326.24		T.cer_06039-	Tmus_05925-RA	Test_02094-		
		PF00326.24		T.cer_02594-RA		Test_05105-		

	Peptidase_S10	PF00450.25	KKA31040.1			Test_00210-		T.pup_05100
	Peptidase_S10	PF00450.25	KKA26396.1					
	Peptidase_S28	PF05577.15	KKA31228.1					
	Peptidase_41	PF03572.21						T.pup_03479
