# Supplementary material for: In Silico Characterization of the Secretome of the Fungal Pathogen Thielaviopsis punctulata, the Causal Agent of Date Palm Black Scorch Disease
Source: J Fungi (Basel). 2023 Feb 27;9(3):303. doi: 10.3390/jof9030303 (PMC10051545; doi:10.3390/jof9030303)
Supplement: Supplementary file 1 [file jof-09-00303-s001.zip › Supplementary Table S5.pdf]

Supplementary Table S5. PHI homologs of secretory proteins of *T. punctulata*

Family	Protein id	Enzyme name	PHI-base entry	Gene Name	Species name	Mutant Phenotype
**Cazymes**						
AA9	KKA27328.1	lytic cellulose monooxygenase	PHI:3216	MoCDIP4	Magnaporthe_oryzae	effector_(plant_avirulence_determinant)
AA9	KKA28497.1	lytic cellulose monooxygenase	PHI:3216	MoCDIP4	Magnaporthe_oryzae	effector_(plant_avirulence_determinant)
AA9	KKA25992.1	endo-β-1,4-glucanase	PHI:3216	MoCDIP4	Magnaporthe_oryzae	effector_(plant_avirulence_determinant)
GH05	KKA26778.1	endo-β-1,4-glucanase	PHI:3216	MoCDIP4	Magnaporthe_oryzae	effector_(plant_avirulence_determinant)
GH07	KKA26295.1	cellulose 1,4-beta-cellobiosidase	PHI:3216	MoCDIP4	Magnaporthe_oryzae	effector_(plant_avirulence_determinant)
GH11	KKA30007.1	endo-β-1,4-xylanase	PHI:11606	Vd424Y	Verticillium_dahliae	effector_(plant_avirulence_determinant)
AA1	KKA29108.1	Laccase	PHI:2700	Lac2	Colletotrichum_orbiculare	reduced_virulence
AA1	KKA30055.1	Laccase	PHI:2920	FET3-1__Fet3-1	Colletotrichum_graminicola	reduced_virulence
AA2	KKA29461.1	peroxidase	PHI:5186	MoAPX2	Magnaporthe_oryzae	reduced_virulence
AA5	KKA28638.1	Oxidase with oxygen as acceptor	PHI:5393	GLX	Fusarium_oxysporum	reduced_virulence
GH03	KKA26832.1	β-glucosidase	PHI:10367	BcBGL3_(BCIN_10g05590)	Botrytis_cinerea	reduced_virulence
GH03	KKA30767.1	β-glucosidase	PHI:10367	BcBGL3_(BCIN_10g05590)	Botrytis_cinerea	reduced_virulence
GH10	KKA29568.1	endo-1,4-β-xylanase	PHI:2204	Endo-1_4-beta-xylanase_[GH10_family]	Magnaporthe_oryzae	reduced_virulence
GH125	KKA28305.1	exo-α-1,6-mannosidase	PHI:785	MGG_04128	Magnaporthe_oryzae	reduced_virulence
GH16	KKA27451.1	licheninase	PHI:6265	Eng1	Histoplasma_capsulatum	reduced_virulence
GT4	KKA26489.1	α-1,6-mannosyltransferase	PHI:9793	VdOCH1	Verticillium_dahliae	reduced_virulence

PL1	KKA26877.1	pectin lyase	PHI:3226	Pnl1	Penicillium_digitatum	reduced_virulence
PL1	KKA27238.1	pectin lyase	PHI:3226	Pnl1	Penicillium_digitatum	reduced_virulence
PL3	KKA30830.1	pectate lyase	PHI:180	PELD	Fusarium_solani	reduced_virulence
AA3	KKA28521.1	Cellobiose dehydrogenase	PHI:11560	B0604	Brucella_melitensis	unaffected_pathogenicity
AA7	KKA27659.1	glucooligosaccharide oxidase	PHI:716	ZEB1	Fusarium_graminearum	unaffected_pathogenicity
AA9	KKA28212.1	lytic cellulose monooxygenase	PHI:1575	GzOB015	Fusarium_graminearum	unaffected_pathogenicity
AA9	KKA25994.1	endo-β-1,4-glucanase	PHI:1575	GzOB015	Fusarium_graminearum	unaffected_pathogenicity
AA9	KKA29219.1	lytic cellulose monooxygenase	PHI:1575	GzOB015	Fusarium_graminearum	unaffected_pathogenicity
CE4	KKA26186.1	chitin deacetylase	PHI:6391	CDA2	Magnaporthe_oryzae	unaffected_pathogenicity
GH05	KKA26007.1	endo-β-1,4-glucanase	PHI:2043	XYL-6	Magnaporthe_oryzae	unaffected_pathogenicity
GH07	KKA28489.1	endo-β-1,4-glucanase	PHI:566	Cel2	Bipolaris_zeicola	unaffected_pathogenicity
GH10	KKA27891.1	endo-1,4-β-xylanase	PHI:4246	Xyl03624	Fusarium_graminearum	unaffected_pathogenicity
GH37	KKA30799.1	α,α-trehalase	PHI:7228	Tre1	Magnaporthe_oryzae	unaffected_pathogenicity
**Proteases**						
C13	KKA30601.1	Carboxy peptidase	PHI:6827	GPI8	Colletotrichum_graminicola	loss_of_pathogenicity
M43	KKA26166.1	Metallo peptidase	PHI:11257	FoMep1	Fusarium_oxysporum	reduced_virulence
M43	KKA27603.1	Metallo peptidase	PHI:2117	SPM1	Magnaporthe_oryzae	reduced_virulence
M43	KKA30997.1	Metallo peptidase	PHI:697	Ugt51E1	Leptosphaeria_maculans	unaffected_pathogenicity
M36	KKA27194.1	Metallo peptidase	PHI:4972	MEP3	Trichophyton_mentagrophytes	unaffected_pathogenicity
M36	KKA29831.1	Metallo peptidase	PHI:5892	FGSG_09382	Fusarium_graminearum	unaffected_pathogenicity
M36	KKA27233.1	Metallo peptidase	PHI:5893	FGSG_10525	Fusarium_graminearum	unaffected_pathogenicity
**Putative effectors**						

	KKA29596.1	**GlcNAc-PI de-N-acetylase**	PHI:6825	GPI12	Colletotrichum_graminicola	loss_of_pathogenicity
	KKA26926.1	Egh16-like	PHI:256	GAS1	Magnaporthe_oryzae	reduced_virulence
	KKA26947.1	Egh16-like	PHI:256	GAS1	Magnaporthe_oryzae	reduced_virulence
	KKA27620.1	CAP superfamily protein	PHI:7144	FvSCP1	Fusarium_verticillioides	reduced_virulence
	KKA27672.1	Egh16-like	PHI:257	GAS2	Magnaporthe_oryzae	reduced_virulence
	KKA25960.1	Exo_endo_phosphatase	PHI:5754		Fusarium graminearum	reduced_virulence
	KKA28484.1	Hypothetical protein	PHI:3209	Pop1	Ceratocystis_harringtonii	reduced_virulence
	KKA28667.1	Spherulation-specific family 4 protein	PHI:10749	CLU5d_(GLRG_04689)	Colletotrichum_graminicola	reduced_virulence
	KKA27537.1	HD-ZIP protein	PHI:8208		Colletotrichum_graminicola	reduced_virulence
	KKA28836.1	cerato-platanin	PHI:9428	SsSm1_(SS1G_10096)	Sclerotinia_sclerotiorum	reduced_virulence
	KKA29410.1	Egh16-like	PHI:256	GAS1	Magnaporthe_oryzae	reduced_virulence
	KKA29465.1	TRP_N protein	PHI:803	MGG_04629	Magnaporthe_oryzae	reduced_virulence
	KKA29708.1	Hypothetical protein	PHI:10749	CLU5d_(GLRG_04689)	Colletotrichum_graminicola	reduced_virulence
	KKA30907.1	Hypothetical protein	PHI:10368	BcBGL4_(BCIN_03g08710)	Botrytis_cinerea	reduced_virulence
	KKA29951.1	S1-P1_nuclease	PHI:10751	CLU5c_(GLRG_04688)	Colletotrichum_graminicola	unaffected_pathogenicity
